# Coley’s Mixed Toxins of Erysipelas and Bacillus Prodigiosus

**Published:** 1912-02

**Authors:** 


COLEY'S MIXED TOXINS OF ERYSIPELAS AND BACILLUS PRODIGIOSUS, Wm. B. Coley of New York, *Surg. Gyn. and Obs.,* August 1911, sums up his experience as follows:

"Up to the present time I have had sixty-five cases of inoperable sarcoma in which the tumors disappeared under the use of the mixed toxins of erysipelas and bacillus prodigiosus. The utmost efforts have been made to trace the after histories of these cases with the following results:

Seven remained alive and well at the end of 15 to 18 years.

Seven remained alive and well at the end of 10 to 15 years.

Seventeen remained alive and well at the end of 5 to 10 years.

Ten remained alive and well at the end of 3 to 5 years.

That is, 41 cases remained well from 3 to 18 years, or 31 from 5 to 17 years."

